# Dynamic Risk Prediction via a Joint Frailty-Copula Model and IPD Meta-Analysis: Building Web Applications

**DOI:** 10.3390/e24050589

**Published:** 2022-04-22

**Authors:** Takeshi Emura, Hirofumi Michimae, Shigeyuki Matsui

**Affiliations:** 1 Biostatistics Center, Kurume University, Kurume 830-0011, Japan; 2 Research Center for Medical and Health Data Science, The Institute of Statistical Mathematics, Tokyo 190-8562, Japan; smatsui@med.nagoya-u.ac.jp; 3 Department of Clinical Medicine (Biostatistics), School of Pharmacy, Kitasato University, Tokyo 108-8641, Japan; michimaeh@pharm.kitasato-u.ac.jp; 4 Department of Biostatistics, Nagoya University Graduate School of Medicine, Nagoya 466-8550, Japan

**Keywords:** clustered data, copula, frailty model, meta-analysis, risk prediction, Shiny, survival analysis

## Abstract

Clinical risk prediction formulas for cancer patients can be improved by dynamically updating the formulas by intermediate events, such as tumor progression. The increased accessibility of individual patient data (IPD) from multiple studies has motivated the development of dynamic prediction formulas accounting for between-study heterogeneity. A joint frailty-copula model for overall survival and time to tumor progression has the potential to develop a dynamic prediction formula of death from heterogenous studies. However, the process of developing, validating, and publishing the prediction formula is complex, which has not been sufficiently described in the literature. In this article, we provide a tutorial in order to build a web-based application for dynamic risk prediction for cancer patients on the basis of the R packages *joint.Cox* and *Shiny*. We demonstrate the proposed methods using a dataset of breast cancer patients from multiple clinical studies. Following this tutorial, we demonstrate how one can publish web applications available online, which can be manipulated by any user through a smartphone or personal computer. After learning this tutorial, developers acquire the ability to build an online web application using their own datasets.

## 1. Introduction

Clinicians often wish to predict the risk of death for their patients on the basis of patient-level information such as clinicopathological status and tumor gene expressions. A variety of statistical and machine learning methods have been developed for clinical risk prediction in patients with breast cancer [1,2,3,4,5,6], gastric cancer [7], ovarian cancer [8,9,10], lymphoma [11,12,13], head and neck cancer [14], and mixed cancer types [15,16,17]. The prediction methods for clinicians help in contributing to the development of personalized medicine by allowing patients to consider their future and clinicians to choose an optimal therapy.

Traditional prediction methods are designed to predict survival from the date of the initial treatment (e.g., the first-line treatment). The classical Cox regression model [18,19] and many other methods have been applied to predict the risk of death from the initial treatment (e.g., 5-year survival). However, a patient could ask about the risk of death in case they develop (or do not develop) metastasis after the initial treatment [20]. Clinicians may need *dynamic* prediction results, where the prediction time can be set arbitrarily, e.g., one year after treatment [20,21,22]. Dynamic prediction is informative for clinicians regarding the subsequent stages of treatments (e.g., the timing of second-line treatment or the termination of treatment) [22,23]. The use of intermediate event information has been suggested to construct a risk prediction scheme using dynamic prediction models [10,20,21,22,23,24].

For a prediction model to be clinically valid, the model needs to follow some guidelines such as the TRIPOD statement [25]. Due to the increased accessibility of datasets of individual patient data (IPD) from multiple studies, different countries, and diverse ethnic groups, the TRIPOD statement is on the way to an extension (https://www.tripod-statement.org/clustered/ accessed on 1 April 2022) [26].

A *joint frailty-copula model* for IPD meta-analyses [9] has demonstrated its ability to develop both clinical prediction and surrogate validation models from heterogenous studies [10]. However, the implementation remains technically complex. For clinicians to employ the abilities of the joint frailty-copula model, it is better to implement a web calculator for predicting survival. In addition, there has been a strong demand for IPD meta-analyses in order to validate surrogate endpoints [27,28,29,30,31,32,33,34,35], which is related to the prediction of overall survival (the true endpoint) by its surrogate. In any case, statistical methods for IPD meta-analyses have to account for between-study heterogeneity in the prediction [10,26], estimation [30,31,32,33,36,37,38], and surrogate validation [30,32,33,34,35].

This article presents a template for developing a web application for dynamic risk prediction based on a joint frailty-copula model and IPD meta-analysis. The main computational tools are the R packages *joint.Cox* [39] and *Shiny* [40]. To bridge developers and users, we effectively connected the development of a complex dynamic prediction scheme and its implementation to a patient’s prognosis. We provide a Shiny-based template for transforming a prediction model to a web application. A dataset for breast cancer was used for illustration.

After learning the proposed template, developers have the ability to make an online web application using their own dataset. The resultant applications are accessible to clinicians without knowledge of R and can be manipulated by any user through a smartphone or personal computer.

This article is organized as follows. Section 2 reviews the background of this research. Section 3 proposes validation methods for dynamic prediction. Section 4 proposes a template for developing a web application with a step-by-step tutorial. Section 5 reports the results of developing a web-based risk prediction tool following the proposed template with a dataset of breast cancer patients. Section 6 concludes with the discussion.

## 2. Background

### 2.1. Review on Dynamic Prediction

**Figure 1:**
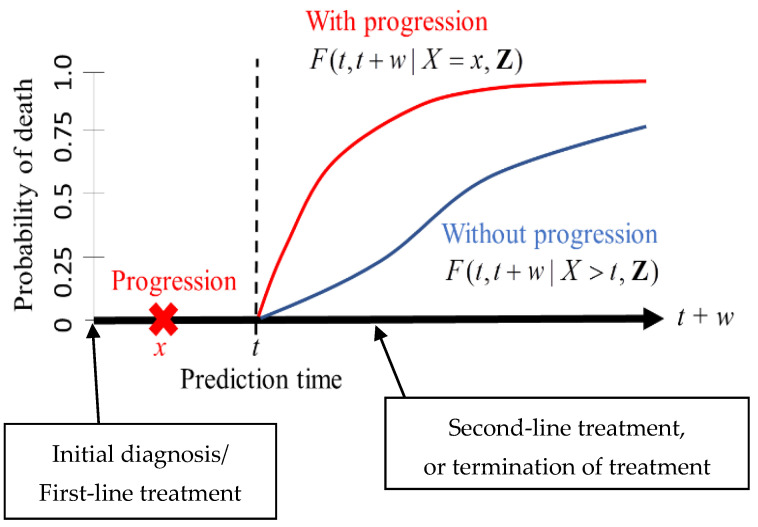
A risk prediction scheme in the framework of dynamic prediction. The measure of prediction is the conditional probability of death between *t* and *t + w* given the observed status of a patient at time *t*. The expressions for *F* are defined in Section 2.3.

We adopted the framework of *dynamic prediction* so that a clinician can set a prediction time point for a patient after the initial diagnosis (or the first-line treatment) [10,20,21,22,23,24]. For instance, a clinician can assess the risk of death for a patient who remains disease-free in the next *t* years after the initial diagnosis or a patient who develops tumor progression within the next *t* years after the diagnosis (Figure 1). The risk assessment at a certain point of time may be informative for clinicians to make decisions on second-line treatments or on the termination of treatment.

The measure of prediction is the conditional probability of death between t and t+w given the observed status of a patient at time t (Figure 1). For instance, one can set t=2 (years) and w=5 (years) when predicting the risk of death within five years for a patient who has survived two years. An important feature of dynamic prediction is the flexibility of choosing a prediction time t at which clinicians can utilize the observed tumor progression status before time t (Figure 1). A statistical model has to be imposed to derive the mathematical expressions of the conditionally predicted probabilities; the details are presented below.

### 2.2. Review on a Joint Frailty-Copula Model

To derive a risk prediction scheme from IPD from multiple studies, we utilized a joint frailty-copula model describing the dependence between time to tumor progression (TTP) and overall survival (OS) [9,10,28,29,30]. The joint frailty-copula model differs from the standard joint models for longitudinal measurements [20,22]. The former is tailored to IPD meta-analyses of multiple studies, where frailty accounts for the between-study heterogeneity, and a copula accounts for the dependence between two event times (TTP and OS). The joint frailty-copula model is especially useful for fitting IPD datasets for validating surrogate endpoints of OS in the meta-analytic framework [28,29,30].

An IPD meta-analysis provides a dataset from G studies with the i-th study containing $N_{i}$ patients for i=1, 2, …, G. Let $X_{ij}$ be TTP and $D_{ij}$ be OS for i=1, 2, …, G and $j=1,\;2,\;\ldots ,\;N_{i}$. Additionally, let $Z_{1ij}$ and $Z_{2ij}$ be covariates for TTP and OS, respectively. Both $X_{ij}$ and $D_{ij}$ can be censored administratively at censoring time $C_{ij}$; $X_{ij}$ can also be censored by death at time $D_{ij}$. What we observe in the dataset are the first-occurring event time $T_{ij}=\min(X_{ij},\;D_{ij},\;C_{ij})$, the status of tumor progression $\delta_{ij}=I\left(T_{ij}=X_{ij}\right)$, the terminal event time $T_{ij}^{*}=\min(D_{ij},\;C_{ij})$, and the status for death $\delta_{ij}^{*}=I\left(T_{ij}^{*}=D_{ij}\right)$, where I(⋅) is the indicator function. Thus, the observed data are expressed as $\left(T_{ij},\;T_{ij}^{*},\;\delta_{ij},\;\delta_{ij}^{*},Z_{1ij},Z_{2ij}\right)$ for i=1, 2, …, G and $j=1,\;2,\;\ldots ,\;N_{i}$.

To model between-study heterogeneity, we introduced “frailty terms”, defined by unobserved positive numbers: $u_{i}$, i=1, 2, …, G [9,10,27,28,29,30,31,32,33,34]. All the patients within the *i*-th study are assumed to have the common risk value $u_{i}$. Conditional on the value $u_{i}$, the risk of each patient is defined by the hazard function for TTP and OS, denoted, respectively, as $r_{ij}\left(t|u_{i},Z_{1ij}\right)=P\left(X_{ij}\leq t+dt|X_{ij}\geq t,u_{i},Z_{1ij}\right)/dt$ and $\lambda_{ij}\left(t|u_{i},Z_{2ij}\right)=P\left(D_{ij}\leq t+dt|D_{ij}\geq t,u_{i},Z_{2ij}\right)/dt$. Additionally, conditional dependence between OS and TTP is modeled by the joint survival function, $P\left(X_{ij}>x,D_{ij}>y|u_{i},Z_{2ij}\right)$.

The joint frailty-copula model [9] specifies hazard functions and the joint survival function in the following form:

$$\left\{\begin{array}{cc} r_{ij}\left(t|u_{i},Z_{1ij}\right)=u_{i}\;r_{0}\left(t\right)\exp\left(\beta_{1}^{'}Z_{1ij}\right); & \mathrm{the hazard function for TTP},\\ \lambda_{ij}\left(t|u_{i},Z_{2ij}\right)=u_{i}^{\alpha }\lambda_{0}\left(t\right)\exp\left(\beta_{2}^{'}Z_{2ij}\right); & \mathrm{the hazard function for OS},\\ C_{\theta }\left[\exp\left\{-u_{i}R_{0}\left(x\right)\exp\left(\beta_{1}^{'}Z_{1ij}\right)\right\},\;\exp\left\{-u_{i}^{\alpha }\Lambda_{0}\left(y\right)\exp\left(\beta_{2}^{'}Z_{2ij}\right)\right\}\right] & \mathrm{the joint survival function}, \end{array}\right.$$

where $\beta_{\cdot }$ is a vector of regression coefficients, $\left(r_{0}\left(\cdot \right),\;\lambda_{0}\left(\cdot \right)\right)$ are the baseline hazard functions, $\left(R_{0}\left(\cdot \right),\;\Lambda_{0}\left(\cdot \right)\right)$ are the cumulative baseline hazard functions, and $C_{\theta }$ is a copula with parameter θ. The frailty term $u_{i}>0$ represents the *i*-th study’s baseline risk not explained by patient-level covariates, assumed to be gamma-distributed with E(u)=1 and Var(u)=η>0, where η represents the degree of heterogeneity [9,27,28,29,30]. The parameter α∈(−∞, ∞) differentiates the effects of u between TTP and OS. Special cases are the null effect on OS (α=0) and the shared effects between TTP and OS (α=1).

For computational simplicity, we particularly adopted the Clayton copula

$$C_{\theta }\left(v,\;w\right)={\left(v^{-\theta }+w^{-\theta }-1\right)}^{-1/\theta },\;\;\theta >0,$$

where θ specifies the dependence between TTP and OS [9,28,29] and can be rescaled into Kendall’s tau: τ=θ/(θ+2). Under the Clayton copula, the presence of tumor progression leads to a (θ+1)-fold higher risk of death [10,28] than the absence of it. Thus, (θ+1) is a relative risk parameter.

All the parameters in the joint frailty-copula model must be estimated by fitting a training dataset. The main advantage of IPD meta-analyses with the joint frailty-copula model is the ability to accommodate a large number of patients collected across different counties and ethnic groups. A model developed from mixed populations is often more precise than a model from a single population [26].

Based on the observed data, one can obtain the parameter estimates $\left(\hat{\alpha },\;\hat{\theta },\;\hat{\eta },\;{\hat{\beta }}_{1},\;{\hat{\beta }}_{2},\;{\hat{r}}_{0},\;{\hat{\lambda }}_{0}\right)$ under the Clayton copula through the *joint.Cox* R package [28]. This package implements a penalized likelihood estimation method using the five-parameter spline for the baseline hazard functions:

$${\hat{r}}_{0}\left(t\right)=\sum_{\ell =1}^{5}\hat{g_{\ell }}M_{\ell }\left(t\right),\;\;\;{\hat{\lambda }}_{0}\left(t\right)=\sum_{\ell =1}^{5}\hat{h_{\ell }}M_{\ell }\left(t\right),\;$$

where $M_{1}\left(t\right),\;\ldots ,\;M_{5}\left(t\right)$ are the M-spline basis functions defined by [9,28], and $\hat{g_{\ell }}>0$ and $\hat{h_{\ell }}>0$ are estimated parameters.

### 2.3. Dynamic Prediction under the Joint Frailty-Copula Model

Dynamic prediction is formulated by the *conditional failure function* [21]. This function is the conditional probability of death between t and t+w given that a patient remains alive at t. Under the joint frailty-copula model, the conditional failure function separates into two cases [10]: First, if a patient does not experience tumor progression before t, the prediction formula is defined as

F(t,t+w| X>t, Z)≡P(D≤t+w|D>t, X>t, Z),

where $Z\equiv \left(Z_{1},Z_{2}\right)$ are covariates for a patient. Second, if a patient experiences tumor progression before t, the prediction formula is defined as

F(t,t+w| X=x, Z)≡P(D≤t+w|D>t, X=x, Z), x≤t.

The two formulas may be combined into one formula by writing F(t,t+w|H(t,X), Z), where H(t,X) represents the tumor status of the patient.

The computation of F(t,t+w|⋅) proceeds as follows: First, we fit a training dataset to the joint frailty-copula model to obtain the parameter estimates $\left(\hat{\alpha },\;\hat{\theta },\;\hat{\eta },\;{\hat{\beta }}_{1},\;{\hat{\beta }}_{2},\;{\hat{r}}_{0},\;{\hat{\lambda }}_{0}\right)$. Next, we compute the survival functions ${\hat{S}}_{X}(t|u)=\exp\left\{-u{\hat{R}}_{0}\left(t\right)\exp\left({\hat{\beta }}_{1}^{\prime }Z_{1}\right)\right\}$ and ${\hat{S}}_{D}(t|u)=\exp\left\{-u^{\alpha }{\hat{\Lambda }}_{0}\left(t\right)\exp\left({\hat{\beta }}_{2}^{\prime }Z_{2}\right)\right\}$. Finally, we compute

(1) $$\hat{F}\left(t,t+w|\;X>t,\;Z\right)=\frac{\int_{\;0}^{\;\infty }\left(C_{\hat{\theta }}[{\hat{S}}_{X}(t|u),\;{\hat{S}}_{D}(t|u)]-C_{\hat{\theta }}[{\hat{S}}_{X}(t|u),\;{\hat{S}}_{D}(t+w|u)]\right)f_{\hat{\eta }}\left(u\right)du}{\int_{\;0}^{\;\infty }C_{\hat{\theta }}[{\hat{S}}_{X}(t|u),\;{\hat{S}}_{D}(t|u)]f_{\hat{\eta }}\left(u\right)du},\;\hat{F}(t,t+w|\;X=x,\;Z)=\frac{\int_{\;0}^{\;\infty }\left(C_{\hat{\theta }}^{\left[0,1\right]}[{\hat{S}}_{X}(x|u),\;{\hat{S}}_{D}(t|u)]-C_{\theta }^{\left[1,0\right]}[{\hat{S}}_{X}(x|u),\;S_{D}(t+w|u)]\right)\;{\hat{r}}_{X}(x|u){\hat{S}}_{X}(x|u)f_{\hat{\eta }}\left(u\right)du}{\int_{\;0}^{\;\infty }C_{\hat{\theta }}^{\left[1,0\right]}[{\hat{S}}_{X}(x|u),\;{\hat{S}}_{D}(t|u)]\;{\hat{r}}_{X}(x|u){\hat{S}}_{X}(x|u)f_{\hat{\eta }}\left(u\right)du},$$

where ${\hat{r}}_{X}(x|u)=-\partial \left\{\log{\hat{S}}_{X}\left(x|u\right)\right\}/\partial x$ and $C_{\theta }^{\left[1,0\right]}\left(v,w\right)=\partial C_{\theta }^{\left[1,0\right]}\left(v,w\right)/\partial v$.

We implemented the computation of $\hat{F}(t,t+w|.,\;Z)$ in the R package *joint.Cox*. The 95% confidence interval (CI) for $\hat{F}(t,t+w|.,\;Z)$ can be obtained via the Monte Carlo method described in Appendix B of [10]. We suggest a graphical display for the 95% CI to visualize the interval length.

### 2.4. Online Web Applications

In a series of papers, Fournier et al. [41], Asar et al. [42], and Lenain et al. [43] developed and validated a web application for dynamic risk predictions of kidney graft failure, making it available online (https://shiny.idbc.fr/DynPG/ accessed on 1 April 2022). Their prediction formula employed a simplified joint model of longitudinal measurements and time to graft failure. To our knowledge, their study was the only one to provide a validated web application for dynamic risk prediction. Following their study, but adopting a different model (the joint frailty-copula model), we provide statistical validation methods and a tutorial to make a web application for developers (Section 3 and Section 4). Based on this tutorial, we made two web applications for predicting overall survival for breast cancer (https://takeshi.shinyapps.io/Breast-2022-0218/ accessed on 1 April 2022) and ovarian cancer (https://takeshi.shinyapps.io/Ovarian-2022-0218/ accessed on 1 April 2022).

## 3. Validation Methods

We propose three statistical methods to validate the dynamic prediction formulas (of Section 2.3), which have not been sufficiently discussed in the context of the joint frailty-copula model.

### 3.1. Calibration Plot

To assess the performance of the dynamic prediction formulas, we propose a calibration plot that measures the agreement between the observed outcomes and predictors [26]. However, the calibration plot as recommended by the TRIPOD statement for non-dynamic formulas under the Cox model [25,26] does not fit our goal. Below, we define a calibration plot for dynamic prediction under the joint frailty-copula model.

For a patient alive at t with his/her tumor status H(t,X) and covariates Z, the survival outcome I(D>t+w) is calibrated by $\hat{S}\left(t,t+w|H\left(t,X\right),\;Z\right)=1-\hat{F}(t,t+w|H\left(t,X\right),\;Z)$. The aim of the calibration plot is to graphically show the agreement between the outcome and predictor based on the observed patients, namely, $I(T_{ij}^{*}>t+w)$ and $\hat{S}\left(t,t+w|H\left(t,T_{ij}\right),\;Z_{ij}\right)$. However, $I(T_{ij}^{*}>t+w)$ is biased for $I(D_{ij}>t+w)$ since $T_{ij}^{*}$ is the censored version of $D_{ij}$. Thus, the following weight function [10,18,19] must be defined for the bias correction:

$${\hat{w}}_{ij}\left(t,\;t+w\right)=\frac{\delta_{ij}^{*}\hat{G}\left(t\right)}{\hat{G}\left(T_{ij}^{*}\right)}I\left(T_{ij}^{*}\leq t+w\right)+\frac{\hat{G}\left(t\right)}{\hat{G}\left(t+w\right)}I\left(T_{ij}^{*}>t+w\right),$$

where $\hat{G}\left(t\right)$ is the Kaplan–Meier (KM) estimate of the censoring survival function $G\left(t\right)=P(C_{ij}>t)$ by treating $T_{ij}^{*}$ as the event time and $1-\delta_{ij}^{*}$ as the event indicator.

We define the observed survival probability:

$$\mathrm{Obs}\;\left(w\right)=\frac{1}{Y\left(t\right)}\underset{ij}{\sum }I(T_{ij}^{*}>t){\hat{w}}_{ij}\left(t,t+w\right)I(T_{ij}^{*}>t+w).$$

We also define the predicted survival probability:

(2) $$\mathrm{Pred}\;\left(w\right)=\frac{1}{Y\left(t\right)}\underset{ij}{\sum }I(T_{ij}^{*}>t){\hat{w}}_{ij}\left(t,t+w\right)\hat{S}(t,t+w|H\left(t,T_{ij}\right),\;Z_{ij}),$$

where $Y\left(t\right)=\sum_{ij}I(T_{ij}^{*}>t)$. We finally define a calibration plot by points $\left\{\mathrm{Pred}\;\left(w_{k}\right),\;\mathrm{Obs}\;\left(w_{k}\right)\right\},\;k=1,2,\ldots ,K$, where $0<w_{1}<\ldots <w_{K}$ is a prespecified sequence. If the plot is placed on the diagonal line defined by $\mathrm{Pred}\;\left(w_{k}\right)=\;\mathrm{Obs}\;\left(w_{k}\right)$, the ideal performance of the prediction formula is achieved.

### 3.2. Brier Score

A good calibration is not enough to show the effectiveness of the dynamic prediction formula. For instance, the dynamic KM estimator $\hat{F}\left(t,t+w\right)=1-{\hat{S}}^{\mathrm{KM}}\left(t+w\right)/{\hat{S}}^{\mathrm{KM}}\left(t\right)$ may exhibit a good calibration plot, where ${\hat{S}}^{\mathrm{KM}}\left(.\right)$ is calculated by partial data $\left(T_{ij}^{*},\;\delta_{ij}^{*}\right)$ for i=1, 2, …, G and $j=1,\;2,\;\ldots ,\;N_{i}$. However, this does not mean that the resultant prediction is precise and efficient. Therefore, to assess the performance of the prediction formula, Emura et al. [10] utilized the Brier scores [44] for the joint model and KM estimator defined, respectively, by

$${\hat{E}\mathrm{rr}}^{\mathrm{JM}}\left(t,t+w\right)=\frac{1}{Y\left(t\right)}\underset{ij}{\sum }I(T_{ij}^{*}>t){\hat{w}}_{ij}\left(t,t+w\right){\left\{I(T_{ij}^{*}>t+w)-\hat{S}(t,t+w|H\left(t,T_{ij}\right),\;Z_{ij})\right\}}^{2},\;{\hat{E}\mathrm{rr}}^{\mathrm{KM}}\left(t,t+w\right)=\frac{1}{Y\left(t\right)}\underset{ij}{\sum }I(T_{ij}^{*}>t){\hat{w}}_{ij}\left(t,t+w\right){\left\{I(T_{ij}^{*}>t+w)-{\hat{S}}^{\mathrm{KM}}\left(t+w\right)/{\hat{S}}^{\mathrm{KM}}\left(t\right)\right\}}^{2}.$$

The confidence interval of the Brier score can be obtained by a bootstrap method [10]. While the Brier score is one of many scoring methods for assessing the prediction performance [45], it is the most commonly used measure in joint models.

We require ${\hat{E}\mathrm{rr}}^{\mathrm{JM}}\left(t,t+w\right)<{\hat{E}\mathrm{rr}}^{\mathrm{KM}}\left(t,t+w\right)$. This requirement may not be trivially satisfied because the dynamic KM estimator itself is a good predictor that is superior to the non-dynamic KM estimator [23]. Therefore, we suggest using a joint model for prediction only if the upper limit of the 95% CI of ${\hat{E}\mathrm{rr}}^{\mathrm{JM}}\left(t,t+w\right)$ is less than ${\hat{E}\mathrm{rr}}^{\mathrm{KM}}\left(t,t+w\right)$.

### 3.3. The C-Index for Discrimination Performance

We propose a method to calculate a dynamic version of the *c*-index. First, as our prediction shall be made for all individuals who are at risk at prediction time t, we define a risk set $R\left(t\right)=\{\left(i,j\right);\;T_{ij}^{*}>t\}$. Next, as our prediction horizon is up to t+w, we define a censored outcome, $\left\{\min\left(T_{ij}^{*},t+w\right),\delta_{ij}^{*}I\left(T_{ij}^{*}\leq t+w\right)\right\}$, for (i,j)∈R(t). The *c*-index is defined as the concordance measure between this outcome and its predictor $\hat{S}(t,t+w|H\left(t,T_{ij}\right),\;Z_{ij})$ given t and t+w. To compute the *c*-index, one can use the R function “concordance(.)” in the survival package.

## 4. Tutorial: Building Web Applications

The implementation of the dynamic prediction tools with the joint frailty-copula model is a highly technical and complex process. Therefore, we present the roadmap for developers who wish to build a web application to implement the prediction method using the R packages *joint.Cox* and *Shiny*. The resultant web application is accessible to users, including clinicians and patients, without knowledge of R.

The proposed methods consist of the following major steps:

**Step 1**:  Fit a training dataset to a joint frailty-copula model using the R package *joint.Cox*;
**Step 2**:  Validate the fitted model;
**Step 3**:  Use the “app.R” file to build a web application using the R package *Shiny*.

In Step 1, a developer obtains a training dataset and fits it to the joint frailty-copula model that is defined in Section 2.2. Step 2 validates the model by checking the prediction capability using the methods presented in Section 3. If the validation results are not satisfactory, the developer goes back to Step 1. If the model is satisfactorily validated, it is transformed to a web application in Step 3. An example of the “app.R” file in Step 3 is the file “app_breast.R” available in the Supplementary Materials.

**Figure 2:**
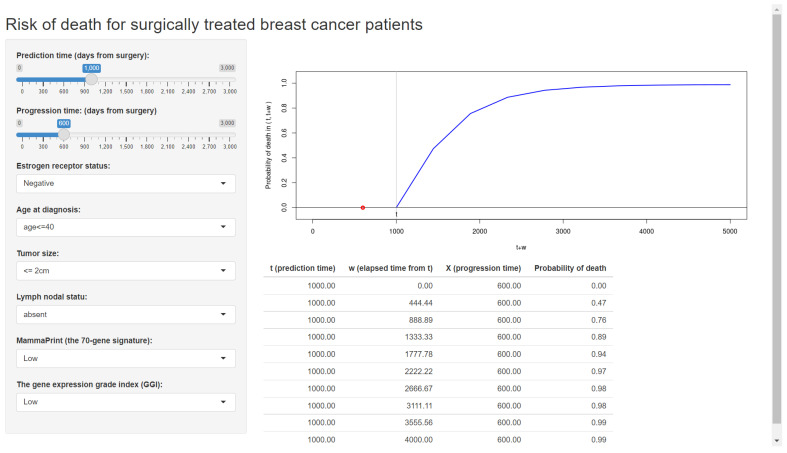
The web application for a clinical prediction tool made by applying the proposed methods to breast cancer data. The interactive version is available at https://takeshi.shinyapps.io/Breast-2022-0218/ (accessed on 1 April 2022).

Figure 2 displays the web application made by Shiny (Step 3), which is already available online.

Steps 1–3 should be performed by developers who have some knowledge of the two R packages. Below are the step-by-step instructions for developers.

**Step 1: Fit a training dataset to a model**.

In this first step, all the unknown parameters in the model have to be estimated by fitting an appropriate training dataset.

We explain this step using the dataset of breast cancer patients from Haibe-Kains et al. [4]. The endpoint of interest is the time to death measured from the date of surgery. From their data, we extracted 720 breast cancer patients having the complete information for the metastasis and death events and their associated covariates (Table 1). All the patients were treated with surgery, and some of them were treated with adjuvant systemic therapy in their first-line treatments.

A developer can fit the joint frailty-copula model to the breast cancer data using the R function *jointCox.reg(.)* and then derive a prediction formula using the R function *F.prediction(.)*. These R functions are the main tools in the R package *joint.Cox*. The R code for applying these functions to the breast cancer data is available in the Supplementary Materials. The usage of the package is detailed in the book of Emura et al. [28] or its preprint available at https://sites.google.com/view/takeshi-emura (accessed on 1 April 2022).

In the joint frailty-copula model, the following covariates should be included:

- Estrogen receptor status (**ER** = 1 for positive; = 0 for negative);
- Tumor size (**Size** = 1 for > 2 cm; = 0 for ≤ 2 cm);
- Lymph nodal status (**Node** = 1 for present; =0 for absent);
- Age at diagnosis (**Age** = 1 for age ≤40; =2 for 40 < age ≤ 50; = 3 for age>50);
- The 70-gene signature developed by [1] (**MammaPrint** = 1 for high; = −1 for low);
- The gene expression grade index (GGI) defined by [3] (**GGI** = 1 for high; = −1 for low).

Briefly, the above covariates were selected by optimizing the likelihood cross-validation (LCV) criterion [28]. The LCV accounts for the number of parameters in the model in a similar manner to the AIC. The details are available upon request to the corresponding author.

**Step 2: Validate the prediction formula.**

Before prediction results are reported to a patient, it is important for a developer to:

(i) Check the confidence interval (CI) for the prediction formula;
(ii) Check the calibration plot, Brier score, and *c*-index.

From (i), one can see the variability of the predicted risk formulas by the length of the CI. A wide CI results from a number of reasons, such as an insufficient number of samples/events and a large value of t or w. Due to its complex nature, it seems unrealistic to determine a sample size requirement formula for the joint frailty-copula model to achieve the required length of the CI. For this reason, the CI is provided only to show the variability of the prediction results, not as a tool to determine the sample size [46].

From (ii), one can assure that the predicted risk is close to the true risk of a patient. Often, a large value of t or w produces a large prediction error [10]. This is because the training dataset may not contain a sufficient amount of observed event times beyond a large value of t. Even if t is small, a larger value of the prediction horizon t+w introduces a larger uncertainty of predicting the occurrence of an event.

Therefore, from (i) and (ii), a developer should choose appropriate values of t and w.

**Step 3: Make a web application using Shiny.**

We suggest the R package *Shiny* to transfer the prediction model (results from Step 1) to a web application. With *Shiny*, one can easily convert R programs into a web application. Usually, this process is carried out by making an “app.R” file, a program file written in the R language. We developed a template file (available in the Supplementary Materials) so that developers can modify the parameters for their own prediction settings. Once the parameters are appropriately tuned by developers according to Steps 1–2, the web application is immediately built by running the template file. The web application can then be made publicly available through a free and self-service platform, *shinyapps.io* (https://www.shinyapps.io/ accessed on 1 April 2022).

## 5. Results

We report the results of developing a web-based risk prediction tool following the tutorial.

The risk prediction tool was developed by fitting the joint frailty-copula model to the dataset of 720 breast cancer patients (Table 1). Each patient yields possibly censored outcomes: time to death and time to metastasis both measured from the date of surgery. These event times were treated as OS and TTP for the joint frailty-copula model. The covariates included in the model are the estrogen receptor status (ER=1 for positive; =0 for negative), tumor size (Size = 1 for >2 cm; = 0 for ≤2 cm), lymph nodal status (Node = 1 for present; =0 for absent), age at diagnosis (Age = 1 for age ≤ 40; = 2 for 40 < age ≤ 50; = 3 for age > 50), MammaPrint (MammaPrint = 1 for high; = −1 for low), and the GGI defined by [3] (GGI = 1 for high; = −1 for low). The R code to reproduce the results of this section is available in the Supplementary Materials.

### 5.1. Model Fitting

We fitted the joint model to the breast cancer data using the R function *jointCox.reg(.)*. Consequently, we obtained the covariate effects on the hazard for time to metastasis and the covariate effects on the hazard for OS.

$$\beta_{1}^{\prime }Z_{1}=(-0.14\times \mathrm{Age})+(-0.23\times \mathrm{ER})+(0.27\times \mathrm{Size})+(0.20\times \mathrm{MammaPrint})+\left(0.19\times \mathrm{GGI}\right)\;\beta_{2}^{\prime }Z_{2}=(-0.36\times \mathrm{ER})+(0.14\times \mathrm{Node})+(0.27\times \mathrm{Size})+(0.17\times \mathrm{MammaPrint})+\left(0.25\times \mathrm{GGI}\right).$$

All the regression coefficients were significant (*p* < 0.05). The estimate of the frailty variance was η=0.067, and its coefficient was α=1, implying the presence of unobserved factors affecting both metastasis and death. The estimate of the Clayton copula parameter was θ=10.7 (95% CI: 8.6–13.4), showing strong dependence between time to metastasis and time to death (Kendall’s tau = 0.84). The baseline hazard functions were estimated as

$${\hat{r}}_{0}\left(t\right)=0.20\times M_{1}\left(t\right)+0.39\times M_{2}\left(t\right)+0.20\times M_{3}\left(t\right)+0.42\times M_{4}\left(t\right)+0.24\times M_{5}\left(t\right),\;{\hat{\lambda }}_{0}\left(t\right)=0.05\times M_{1}\left(t\right)+0.38\times M_{2}\left(t\right)+0.37\times M_{3}\left(t\right)+0.09\times M_{4}\left(t\right)+0.00\times M_{5}\left(t\right),$$

for t<9108 (days), which is the maximum follow-up date, where the *M*’s are the spline basis functions [28]. More details on the model fitting processes are available upon request.

### 5.2. Developing and Validating a Predictor

To develop and validate the prediction formula, we considered a hypothetical patient:

**Patient 1:**

- Age at diagnosis: 45 years
- Estrogen receptor: positive
- Tumor size: >2cm
- Lymph nodal status: present
- MammaPrint: high
- GGI: high

There are 23 breast cancer patients (out of 720 patients) with the same covariate status as Patient 1. Among them, 5 patients experienced metastasis within their follow-up period (the other 18 patients were censored, and thus their metastatic status is unknown). Among the five patients, two developed metastasis within 1000 days, and the other three developed metastasis after 1000 days. Accordingly, we set the prediction time at t=1000 (days) and then considered two scenarios: (a) Patient 1 does not develop metastasis by 1000 days; (b) Patient 1 develops metastasis at 300 days.

**Figure 3:**
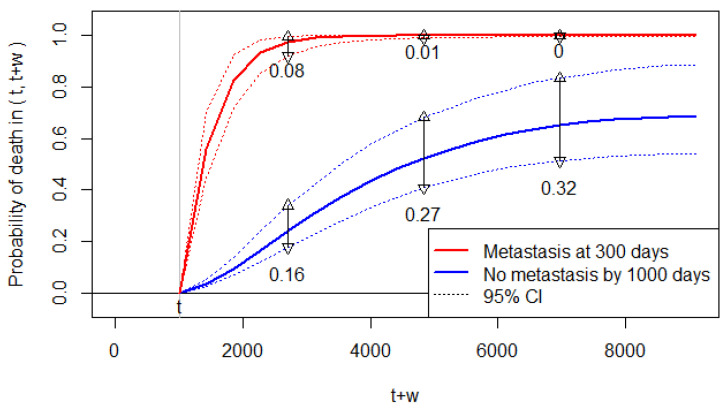
The predicted probability of death when the prediction time is set at *t* = 1000 days. The 95% CIs are indicated by the dotted lines (……), and their widths are shown by the vertical lines (and the number below the lines) at three time points.

Accordingly, Figure 3 displays the conditional probability of death predicted at t=1000 (days) for 0≤w≤8108, which is the allowable range of w for this dataset; the figure displays F(1000,1000+w|X>t, Z) for scenario (a), and F(1000,1000+w|X=300, Z) for scenario (b). We can observe that the predicted risk of death increases significantly by the metastasis at 300 days.

Below, we validate the prediction formulas by checking the CI, calibration plot, Brier score, and *c*-index.

The 95% CIs for the predicted probabilities are sufficiently narrow to discriminate the two scenarios. However, the widths of the 95% CI are somewhat wide for the predicted probabilities without metastasis (Figure 3). Thus, while we are certain about the increased risk of death by metastasis, the predicted probabilities of death have some uncertainty for Patient 1 with no metastasis. For Patient 1 with metastasis at 300 days, the predicted probabilities almost reach the upper bound of one. Thus, we are almost certain about the future death of Patient 1 if she/he develops metastasis.

Note that Figure 3 shows the results under a specific prediction setting. A clinician may need a global risk measure for the increase in death after metastasis without specifying the prediction setting. One way is to report the relative risk parameter estimated as (θ+1)=11.7 (95% CI: 9.6–14.4). Thus, a patient with metastasis has an 11.7 times higher risk of death compared to a patient without metastasis.

**Figure 4:**
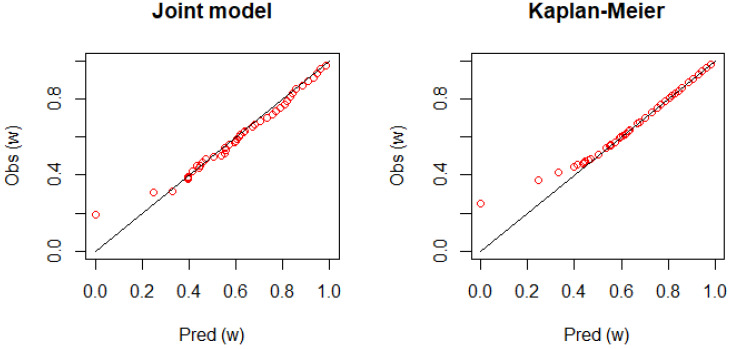
The calibration plots comparing the observed and predicted survival rates, consisting of $\left\{\mathrm{Pred}\;\left(w_{k}\right),\;\mathrm{Obs}\;\left(w_{k}\right)\right\}$ with equally spaced prediction horizons, $0<w_{1}<\ldots <w_{50}=8108$ (days). If the plots are placed on the diagonal line, the ideal performance of the prediction formula is achieved. **Left panel**: the joint frailty-copula model; **right panel**: the dynamic KM estimator.

Figure 4 shows the calibration plot for Patient 1. The figure displays a good agreement between the observed survival rate and predicted survival rate as the plot is placed on the diagonal line. However, it is not clear how the joint model performs better than the dynamic KM estimator.

**Figure 5:**
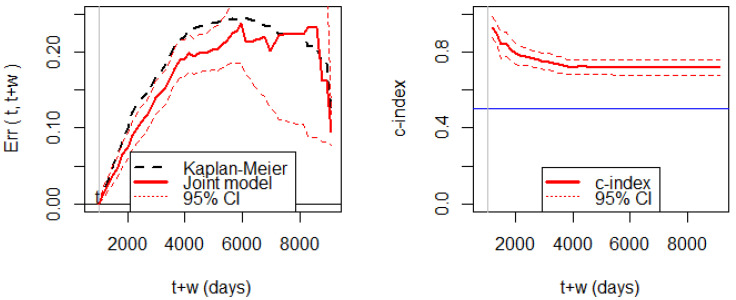
**(Left panel**): prediction errors (Brier score) based on the breast cancer data at the prediction time at 1000 days. (**Right panel**): the *c*-index for discrimination ability with the 95% CI based on the same setting.

Figure 5 shows the Brier score for assessing prediction errors. We can observe that the prediction error of the joint model is significantly smaller than the prediction error of the KM estimator before 5000 days. The wide width of the 95% CI after 5000 days is due to the small number of patients alive. The *c*-index shows that the discrimination ability is consistently higher than 0.5.

Hence, the joint model has enough predictive power up to 5000 days, though the maximum follow-up time is 9108 days. In other words, a reliable prediction is not possible beyond 5000 days. Therefore, when we set t=1000 (days), we have to choose the prediction horizon by 0≤t+w≤5000. Thus, 0<w≤4000 is the allowable range of w for this dataset and the joint model.

### 5.3. Upload a Web Application

On the basis of the developed prediction formula for breast cancer patients, we wrote a program file that can run under the *Shiny* package [40]. This file builds a web application and also publishes the application on the internet. For reference for users, we provide a template file, “app_breast”, in the Supplementary Materials, which can be directly used to upload our web application on the internet. With this file, we converted the developed prediction formula into a web application and published it online (Figure 2).

The concrete steps to build an online application are as follows.

**Step 1**:  Open the “app_breast” file in R studio;
**Step 2**:  Run the code in the file (as with the usual R code), and then the application is generated in a window;
**Step 3**:  Check if the application works properly;
**Step 4**:  Publish the application (click the “Publish” icon).

Developers may edit our template file “app_breast” according to their training data. Developers working on their own data need to add/delete input variables and adjust all the model parameters and prediction time points. The editing process requires the basic programing skills for R and *Shiny*.

**Figure 6:**
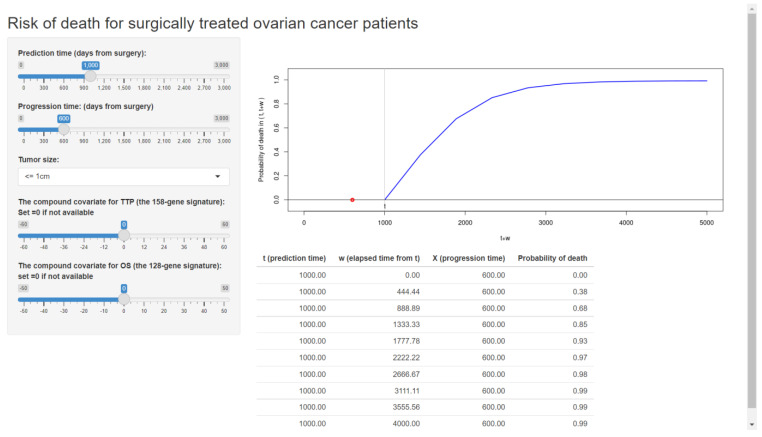
The web application for a clinical prediction tool made by applying the proposed methods to ovarian cancer data. The interactive version is available at https://takeshi.shinyapps.io/Ovarian-2022-0218/ (accessed on 1 April 2022).

As an additional example, we also built our web application based on our previously validated formula for ovarian cancer patients [10,28]. The published web application is shown in Figure 6.

Following the tutorial, developers can publish their original web applications using their own validated prediction formulas for their targeted patients.

## 6. Conclusions and Discussion

In this article, we present a tutorial for developers who wish to build a web application for the personalized risk prediction method of [10]. While we reviewed the methods of [10], we also propose new validation tools: the calibration plot and the *c*-index, that were not considered in the context of the joint frailty-copula model. We provide a Shiny-based template to transfer the prediction model to a user-friendly web application. Following the proposed tutorial and template, we built two web applications for predicting the risk for breast cancer patients (Figure 2) and ovarian cancer patients (Figure 6). Developers learning our tutorial will have the ability to create a variety of online web applications using their own datasets.

According to the predicted risk of death, a clinician may suggest a second-line treatment, or he/she may suggest stopping the treatment [23]. The key factor is whether the clinician regards tumor progression as the failure or the inadequacy of the initial treatment. In this respect, clinicians can make their decision on the second-line treatment with the help of the risk of death with or without tumor progression (Figure 1).

Throughout this article, we used a breast cancer dataset as a demonstrative example for developing a dynamic risk prediction scheme. This is our second example of demonstrating the joint frailty-copula model as a reliable dynamic prediction scheme. Our previous work [10] employed the ovarian cancer dataset of Ganzfried et al. [47] to develop a risk prediction formula of death according to tumor progression status, residual tumor size, and genetic covariates. Other cancer datasets from other clinical studies can be utilized for building web applications, such as a dataset for bladder cancer with an appropriate treatment of competing risks [48,49].

Note that the developed prediction formula for breast cancer patients (Figure 2) has many more clinicopathological covariates than the model for ovarian cancer patients (Figure 6). This is because many of the clinicopathological covariates in ovarian cancer are not significant, nor do they effectively improve the simple model of the residual tumor size alone. The poor predictive power of clinical covariates has motivated a number of survival prediction models with gene expressions in ovarian cancers [8,9,10,50,51]. While the residual tumor size is considered the strongest clinical predictor of survival, the tumor progression information has even stronger predictive ability for overall survival [10], providing a rationale for applying dynamic prediction tools. According to these experiences, the proposed dynamic prediction scheme with the joint model would be promising for other cancers as long as the clinician can properly use it.

In this study, we employed the R package *Shiny* to transfer complex prediction formulas to a user-friendly web application. Recently, Fournier et al. [41], Asar et al. [42], and Lenain et al. [43] made a Shiny-based web application for the dynamic prediction of long-term kidney graft failure. Clearly, this type of dynamic prediction tool can promote the development of personalized medicine, allowing clinicians to utilize powerful prediction algorithms without detailed knowledge of R.

The issue that we did not discuss in this article is the construction of a multigene predictor, an important element in personalized medicine. For the breast cancer dataset, we employed the commercially available prognostic signatures MammaPrint [1] and GGI [3]. If there is no established signature, one often has to apply a gene selection (feature selection) method for a number of available genes. One of the most frequently used methods is to select genes according to the significance levels of the associations between each gene and survival [12,13,52,53,54]. One may apply the *compound.Cox* R package [52] to select an optimal set of genes while assessing their false discovery rate.

The issue of dynamic prediction is not restricted to medical research, which can be seen in the reliability analysis of mechanical items or systems [55,56,57,58,59]. In the reliability prediction of mechanical equipment [56,57,58,59], the conditional failure/survival probability was suggested as a measure of prediction in a similar fashion to our prediction scheme in Figure 1. The present tutorial can be a useful guide for building a web application for these engineering issues. Since statistical models in engineering applications involve time-dependent covariates [58] or dependent component failures [60,61,62], the derivation of a prediction formula is challenging.

This article considers two endpoints (TTP and OS) via the joint frailty-copula model. While more than two endpoints could be observable in clinical trials, the computational cost for implementing multivariate joint models is high, and some approximation techniques are always necessary (see [63,64,65,66,67] and references therein). Currently, the joint frailty-copula model has not been extended to multivariate settings due to its computational difficulty, mainly caused by the need for numerical integrations for frailty terms. Parametric joint frailty-copula models, such as the Weibull and Pareto joint frailty-copula models, reduce the computational cost to some degree [68,69], though they are less flexible compared to spline-based models.

## Figures and Tables

**Table 1:** The breast cancer dataset of Haibe-Kains et al. [4].

Maximum Follow-Up Days	Dataset ^a^	*N*	The Number of Observed Events (Event Rates)		
			Metastasis	Death	Censoring
5165	CAL	109	24 (22%)	75 (69%)	34 (31%)
6694	NKI	295	101 (34%)	79 (27%)	216 (73%)
9108	TRANSBIG	196	62 (32%)	56 (29%)	140 (71%)
8267	UCSF	120	19 (16%)	39 (32%)	81 (68%)
9108	Total	720	206 (29%)	249(35%)	471 (65%)

**Notes:** The R code for obtaining the data is available in the Supplementary Materials. The data are a subset from the file “jnci-JNCI-11-0924-s02.csv” available in the Supplementary Data of Haibe-Kains et al. [4]; the file is available on the journal webpage. ^a^ Datasets are signified as acronyms: CAL = dataset of breast cancer patients from the University of California, San Francisco, and the California Pacific Medical Center (United States); NKI = National Kanker Institute (the Netherlands); TRANSBIG = dataset collected by the TransBIG consortium (Europe); UCSF = University of California, San Francisco (United States). The extracted data are the subset having complete values of “t.dmfs: time for distant metastasis-free survival (days)”, “e.dmfs: event for distant metastasis-free survival”, “t.os: time for overall survival (days)”, and “e.os: event for overall survival”, as well as covariates (ER, Size, Node, Age, MammaPrint, and GGI). The median follow-up time was calculated from the Kaplan–Meier estimator for the time to censoring for each study. The event rates were calculated separately for each study.

## Data Availability

All the numerical results of the article are reproduced by the R code in Supplementary Materials.

## Supplementary Materials

The following supporting information can be downloaded at: https://www.mdpi.com/article/10.3390/e24050589/s1, Supplementary Materials of this article include the following items: File S1. R code to analyze the breast cancer data; File S2. A template file “app_breast. R” for Shiny.
